# Bis[2-(cyclo­pentyl­imino­meth­yl)-4-nitro­phenolato-κ^2^ *N* ^2^,*O*]cobalt(II)

**DOI:** 10.1107/S1600536811002194

**Published:** 2011-01-22

**Authors:** Mai Xu, Yi-Jun Wei, Feng-Wu Wang

**Affiliations:** a Department of Chemistry, Huainan Normal College, Huainan 232001, People’s Republic of China

## Abstract

In the title compound, [Co(C_12_H_13_N_2_O_3_)_2_], the Co^II^ ion is situated on a twofold rotation axis and is coordinated by two N and two O atoms from two symmetry-related Schiff base 2-(cyclo­pentyl­imino­meth­yl)-4-nitro­phenolate ligands (*L*) in a distorted tetra­hedral geometry. The cyclo­pentyl ring in *L* is disordered over two conformations in a 0.640 (19):0.360 (19) ratio.

## Related literature

For background to Schiff bases and their complexes, see: Salehzadeh *et al.* (2010 ▶). For cobalt(II/III) complexes with Schiff base ligands, see: Nejo *et al.* (2010 ▶); Shahabadi *et al.* (2010 ▶). For the Schiff base complexes we have reported, see: Wei *et al.* (2008 ▶); Wang *et al.* (2007 ▶). For similar cobalt(II) complexes with Schiff bases, see: Bahron *et al.* (1994 ▶); Elerman *et al.* (1996 ▶).
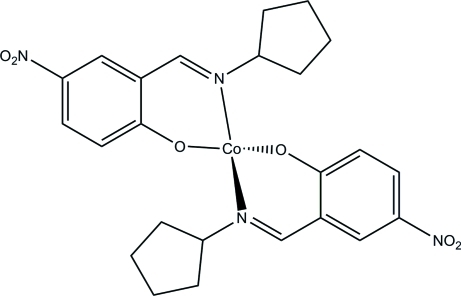


## Experimental

### 

#### Crystal data

[Co(C_12_H_13_N_2_O_3_)_2_]
*M* *_r_* = 525.42
Orthorhombic, 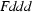

*a* = 18.057 (2) Å
*b* = 18.792 (2) Å
*c* = 30.070 (4) Å
*V* = 10203 (2) Å^3^
*Z* = 16
Mo *K*α radiation
μ = 0.72 mm^−1^
*T* = 298 K
0.17 × 0.13 × 0.12 mm

#### Data collection

Bruker SMART CCD area-detector diffractometer
Absorption correction: multi-scan (*SADABS*; Bruker, 2001 ▶) *T*_min_ = 0.888, *T*_max_ = 0.919
13092 measured reflections
2381 independent reflections
1143 reflections with *I* > 2σ(*I*)
*R*_int_ = 0.099

#### Refinement

*R*[*F* ^2^ > 2σ(*F* ^2^)] = 0.054
*wR*(*F* ^2^) = 0.182
*S* = 0.92
2381 reflections
196 parameters
66 restraints
H-atom parameters constrained
Δρ_max_ = 0.67 e Å^−3^
Δρ_min_ = −0.29 e Å^−3^

### 

Data collection: *SMART* (Bruker, 2007 ▶); cell refinement: *SAINT* (Bruker, 2007 ▶); data reduction: *SAINT*; program(s) used to solve structure: *SHELXTL* (Sheldrick, 2008 ▶); program(s) used to refine structure: *SHELXTL*; molecular graphics: *SHELXTL*; software used to prepare material for publication: *SHELXTL*.

## Supplementary Material

Crystal structure: contains datablocks global, I. DOI: 10.1107/S1600536811002194/cv5039sup1.cif

Structure factors: contains datablocks I. DOI: 10.1107/S1600536811002194/cv5039Isup2.hkl

Additional supplementary materials:  crystallographic information; 3D view; checkCIF report

## Figures and Tables

**Fig. 1.**
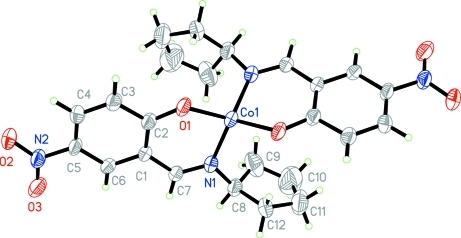
Molecular structure of the title compound showing the atomic numbering and 30% probability displacement ellipsoids. Unlabeled atoms are related with the labeled ones by symmetry operation (3/4 - x, 7/4 - y, z). Only major parts of the disordered cyclopentyl rings are shown.

## supplementary crystallographic information

### Comment

The condensation reaction of aromatic carbaldehydes with primary amines has been shown to offer an easy and inexpensive way of forming a variety of polydentate Schiff base ligands (Salehzadeh *et al.*, 2010). Cobalt(II/III) complexes with Schiff base ligands have been studied extensively due to their interesting structures and wide applications (Nejo *et al.*, 2010; Shahabadi *et al.*, 2010). As a continuation of our work on Schiff base complexes (Wei *et al.*, 2008; Wang *et al.*, 2007), the title mononuclear cobalt(II) complex (Fig. 1) is reported here.

The complex is located on a twofold rotational axis. The tetrahedral coordination sphere of Co^II^ atom in the complex is formed by two imino N atoms and two phenolate O atoms from two Schiff base ligands. The coordinate bond distances are typical and comparable with the values in other similar cobalt(II) complexes with Schiff bases (Bahron *et al.*, 1994; Elerman *et al.*, 1996). The coordinate bond angles are in the range 96.06 (14)–123.0 (2)°, indicating a distorted tetrahedral geometry.

### Experimental

The title complex was obtained by stirring of 5-nitrosalicylaldehyde (0.1 mmol, 16.7 mg), cyclopentylamine (0.1 mmol, 8.5 mg), and cobalt(II) acetate (0.1 mmol, 24.9 mg) in methanol (20 ml) for 30 min at room temperature. The reaction mixture was fitered. Brown block-shaped single crystals suitable for X-ray diffraction were formed from the filtrate after a few days.

### Refinement

Hydrogen atoms were positioned geometrically and refined using the riding-model approximation, with C—H = 0.93–0.98 Å, and *U*_iso_(H) = 1.2*U*_eq_(C). The cyclohexyl ring is disordered over two sites with occupancies of 0.360 (2) and 0.640 (2).

### Crystal data

**Table:**

[Co(C_12_H_13_N_2_O_3_)_2_]	*D*_x_ = 1.368 Mg m^−^^3^
*M**_r_* = 525.42	Mo *K*α radiation, λ = 0.71073 Å
Orthorhombic, *F**d**d**d*	Cell parameters from 1748 reflections
*a* = 18.057 (2) Å	θ = 2.3–24.5°
*b* = 18.792 (2) Å	µ = 0.72 mm^−^^1^
*c* = 30.070 (4) Å	*T* = 298 K
*V* = 10203 (2) Å^3^	Block, brown
*Z* = 16	0.17 × 0.13 × 0.12 mm
*F*(000) = 4368	

### Data collection

**Table:**

Bruker SMART CCD area-detector diffractometer	2381 independent reflections
Radiation source: fine-focus sealed tube	1143 reflections with *I* > 2σ(*I*)
graphite	*R*_int_ = 0.099
ω scans	θ_max_ = 25.5°, θ_min_ = 1.7°
Absorption correction: multi-scan (*SADABS*; Bruker, 2001)	*h* = −21→18
*T*_min_ = 0.888, *T*_max_ = 0.919	*k* = −20→22
13092 measured reflections	*l* = −29→36

### Refinement

**Table:**

Refinement on *F*^2^	Primary atom site location: structure-invariant direct methods
Least-squares matrix: full	Secondary atom site location: difference Fourier map
*R*[*F*^2^ > 2σ(*F*^2^)] = 0.054	Hydrogen site location: inferred from neighbouring sites
*wR*(*F*^2^) = 0.182	H-atom parameters constrained
*S* = 0.92	*w* = 1/[σ^2^(*F*_o_^2^) + (0.1016*P*)^2^] where *P* = (*F*_o_^2^ + 2*F*_c_^2^)/3
2381 reflections	(Δ/σ)_max_ < 0.001
196 parameters	Δρ_max_ = 0.67 e Å^−^^3^
66 restraints	Δρ_min_ = −0.29 e Å^−^^3^

### Special details

**Table:**

Geometry. All e.s.d.'s (except the e.s.d. in the dihedral angle between two l.s. planes) are estimated using the full covariance matrix. The cell e.s.d.'s are taken into account individually in the estimation of e.s.d.'s in distances, angles and torsion angles; correlations between e.s.d.'s in cell parameters are only used when they are defined by crystal symmetry. An approximate (isotropic) treatment of cell e.s.d.'s is used for estimating e.s.d.'s involving l.s. planes.
Refinement. Refinement of *F*^2^ against ALL reflections. The weighted *R*-factor *wR* and goodness of fit *S* are based on *F*^2^, conventional *R*-factors *R* are based on *F*, with *F* set to zero for negative *F*^2^. The threshold expression of *F*^2^ > σ(*F*^2^) is used only for calculating *R*-factors(gt) *etc*. and is not relevant to the choice of reflections for refinement. *R*-factors based on *F*^2^ are statistically about twice as large as those based on *F*, and *R*- factors based on ALL data will be even larger.

### Fractional atomic coordinates and isotropic or equivalent isotropic displacement parameters (Å^2^)

**Table:**

	*x*	*y*	*z*	*U*_iso_*/*U*_eq_	Occ. (<1)
Co1	0.3750	0.8750	0.69805 (4)	0.0616 (4)	
N1	0.4685 (2)	0.8513 (2)	0.66652 (14)	0.0627 (11)	
N2	0.5105 (3)	0.5346 (2)	0.69096 (17)	0.0724 (13)	
O1	0.36714 (17)	0.78730 (16)	0.72938 (12)	0.0692 (10)	
O2	0.4809 (2)	0.47999 (19)	0.70349 (16)	0.0979 (14)	
O3	0.5686 (3)	0.53537 (19)	0.66859 (15)	0.0857 (12)	
C1	0.4679 (3)	0.7286 (2)	0.69236 (16)	0.0532 (12)	
C2	0.4045 (3)	0.7292 (2)	0.71991 (18)	0.0593 (13)	
C3	0.3787 (3)	0.6643 (3)	0.73778 (18)	0.0673 (14)	
H3	0.3373	0.6643	0.7562	0.081*	
C4	0.4134 (3)	0.6022 (3)	0.72853 (19)	0.0686 (15)	
H4	0.3952	0.5599	0.7403	0.082*	
C5	0.4751 (3)	0.6013 (2)	0.70195 (18)	0.0603 (14)	
C6	0.5035 (3)	0.6637 (3)	0.68422 (16)	0.0601 (13)	
H6	0.5462	0.6624	0.6669	0.072*	
C7	0.4979 (3)	0.7890 (2)	0.67028 (17)	0.0625 (14)	
H7	0.5439	0.7829	0.6570	0.075*	
C8	0.5093 (3)	0.9036 (2)	0.63903 (19)	0.0792 (18)	0.360 (19)
H8A	0.5478	0.8772	0.6231	0.095*	0.360 (19)
C9	0.4576 (10)	0.9360 (6)	0.6035 (5)	0.058 (6)	0.360 (19)
H9A	0.4097	0.9126	0.6038	0.069*	0.360 (19)
H9B	0.4790	0.9313	0.5741	0.069*	0.360 (19)
C10	0.4500 (11)	1.0153 (7)	0.6166 (8)	0.105 (8)	0.360 (19)
H10A	0.4142	1.0219	0.6402	0.127*	0.360 (19)
H10B	0.4363	1.0445	0.5913	0.127*	0.360 (19)
C11	0.5309 (11)	1.0318 (8)	0.6328 (8)	0.084 (8)	0.360 (19)
H11A	0.5651	1.0349	0.6080	0.101*	0.360 (19)
H11B	0.5329	1.0758	0.6497	0.101*	0.360 (19)
C12	0.5479 (14)	0.9666 (7)	0.6625 (8)	0.065 (8)	0.360 (19)
H12A	0.6009	0.9585	0.6645	0.078*	0.360 (19)
H12B	0.5282	0.9733	0.6922	0.078*	0.360 (19)
C8'	0.5093 (3)	0.9036 (2)	0.63903 (19)	0.0792 (18)	0.640 (19)
H8'A	0.5543	0.8822	0.6267	0.095*	0.640 (19)
C9'	0.4605 (12)	0.9347 (7)	0.6014 (6)	0.184 (12)	0.640 (19)
H9'A	0.4088	0.9361	0.6102	0.220*	0.640 (19)
H9'B	0.4651	0.9068	0.5744	0.220*	0.640 (19)
C10'	0.4914 (9)	1.0109 (6)	0.5946 (4)	0.109 (5)	0.640 (19)
H10C	0.4549	1.0420	0.5811	0.131*	0.640 (19)
H10D	0.5359	1.0106	0.5765	0.131*	0.640 (19)
C11'	0.5087 (10)	1.0337 (5)	0.6438 (4)	0.104 (6)	0.640 (19)
H11C	0.5496	1.0671	0.6444	0.124*	0.640 (19)
H11D	0.4657	1.0563	0.6569	0.124*	0.640 (19)
C12'	0.5288 (9)	0.9665 (5)	0.6696 (4)	0.075 (5)	0.640 (19)
H12C	0.5007	0.9639	0.6971	0.090*	0.640 (19)
H12D	0.5811	0.9662	0.6768	0.090*	0.640 (19)

### Atomic displacement parameters (Å^2^)

**Table:**

	*U*^11^	*U*^22^	*U*^33^	*U*^12^	*U*^13^	*U*^23^
Co1	0.0590 (6)	0.0357 (5)	0.0903 (8)	0.0076 (4)	0.000	0.000
N1	0.062 (3)	0.041 (2)	0.086 (3)	0.0031 (19)	0.002 (2)	0.006 (2)
N2	0.080 (3)	0.040 (3)	0.097 (4)	0.007 (2)	−0.019 (3)	0.001 (3)
O1	0.073 (2)	0.0385 (19)	0.097 (3)	0.0136 (17)	0.015 (2)	0.0044 (17)
O2	0.100 (3)	0.038 (2)	0.155 (4)	0.007 (2)	−0.010 (3)	0.001 (2)
O3	0.097 (3)	0.060 (2)	0.100 (3)	0.026 (2)	−0.008 (3)	−0.005 (2)
C1	0.051 (3)	0.038 (3)	0.070 (3)	0.006 (2)	−0.003 (3)	−0.003 (2)
C2	0.063 (3)	0.042 (3)	0.073 (3)	0.007 (2)	−0.014 (3)	0.002 (2)
C3	0.069 (3)	0.044 (3)	0.089 (4)	0.002 (3)	0.011 (3)	0.008 (3)
C4	0.073 (4)	0.040 (3)	0.093 (4)	−0.002 (3)	−0.003 (3)	0.014 (3)
C5	0.065 (3)	0.036 (3)	0.080 (4)	0.009 (2)	−0.014 (3)	−0.002 (2)
C6	0.057 (3)	0.047 (3)	0.077 (4)	0.012 (2)	−0.008 (3)	−0.004 (2)
C7	0.055 (3)	0.044 (3)	0.088 (4)	0.003 (2)	0.007 (3)	0.002 (3)
C8	0.071 (4)	0.048 (3)	0.119 (5)	0.004 (3)	0.024 (3)	0.014 (3)
C9	0.076 (10)	0.050 (8)	0.048 (8)	−0.032 (6)	−0.013 (7)	0.022 (6)
C10	0.101 (11)	0.111 (11)	0.104 (12)	0.025 (8)	0.000 (9)	0.008 (8)
C11	0.089 (11)	0.062 (10)	0.100 (12)	0.003 (7)	0.014 (9)	−0.009 (8)
C12	0.059 (11)	0.052 (10)	0.083 (11)	−0.004 (7)	0.017 (8)	0.001 (7)
C8'	0.071 (4)	0.048 (3)	0.119 (5)	0.004 (3)	0.024 (3)	0.014 (3)
C9'	0.189 (14)	0.174 (14)	0.188 (14)	−0.040 (9)	−0.001 (9)	0.020 (9)
C10'	0.110 (8)	0.093 (7)	0.126 (9)	0.011 (6)	−0.001 (7)	0.037 (6)
C11'	0.120 (10)	0.067 (7)	0.125 (9)	−0.008 (6)	0.024 (8)	0.015 (6)
C12'	0.054 (7)	0.067 (7)	0.105 (8)	−0.022 (5)	0.003 (6)	0.011 (5)

### Geometric parameters (Å, °)

**Table:**

Co1—O1^i^	1.904 (3)	C8—H8A	0.9800
Co1—O1	1.904 (3)	C9—C10	1.548 (10)
Co1—N1^i^	1.987 (4)	C9—H9A	0.9700
Co1—N1	1.987 (4)	C9—H9B	0.9700
N1—C7	1.291 (5)	C10—C11	1.571 (10)
N1—C8	1.480 (6)	C10—H10A	0.9700
N2—O2	1.217 (5)	C10—H10B	0.9700
N2—O3	1.248 (6)	C11—C12	1.549 (10)
N2—C5	1.444 (6)	C11—H11A	0.9700
O1—C2	1.314 (5)	C11—H11B	0.9700
C1—C6	1.401 (6)	C12—H12A	0.9700
C1—C2	1.413 (7)	C12—H12B	0.9700
C1—C7	1.422 (6)	C9'—C10'	1.551 (9)
C2—C3	1.411 (6)	C9'—H9'A	0.9700
C3—C4	1.353 (7)	C9'—H9'B	0.9700
C3—H3	0.9300	C10'—C11'	1.569 (9)
C4—C5	1.371 (7)	C10'—H10C	0.9700
C4—H4	0.9300	C10'—H10D	0.9700
C5—C6	1.387 (7)	C11'—C12'	1.527 (8)
C6—H6	0.9300	C11'—H11C	0.9700
C7—H7	0.9300	C11'—H11D	0.9700
C8—C9	1.544 (9)	C12'—H12C	0.9700
C8—C12	1.545 (10)	C12'—H12D	0.9700
			
O1^i^—Co1—O1	120.7 (2)	C8—C9—H9A	110.8
O1^i^—Co1—N1^i^	96.06 (14)	C10—C9—H9A	110.8
O1—Co1—N1^i^	111.50 (16)	C8—C9—H9B	110.8
O1^i^—Co1—N1	111.50 (16)	C10—C9—H9B	110.8
O1—Co1—N1	96.06 (14)	H9A—C9—H9B	108.8
N1^i^—Co1—N1	123.0 (2)	C9—C10—C11	100.8 (10)
C7—N1—C8	116.5 (4)	C9—C10—H10A	111.6
C7—N1—Co1	120.7 (3)	C11—C10—H10A	111.6
C8—N1—Co1	122.7 (3)	C9—C10—H10B	111.6
O2—N2—O3	123.0 (5)	C11—C10—H10B	111.6
O2—N2—C5	117.9 (5)	H10A—C10—H10B	109.4
O3—N2—C5	119.1 (5)	C12—C11—C10	101.9 (10)
C2—O1—Co1	125.0 (3)	C12—C11—H11A	111.4
C6—C1—C2	118.8 (5)	C10—C11—H11A	111.4
C6—C1—C7	116.0 (5)	C12—C11—H11B	111.4
C2—C1—C7	125.1 (4)	C10—C11—H11B	111.4
O1—C2—C3	117.8 (5)	H11A—C11—H11B	109.3
O1—C2—C1	123.3 (4)	C8—C12—C11	104.7 (9)
C3—C2—C1	118.9 (4)	C8—C12—H12A	110.8
C4—C3—C2	121.0 (5)	C11—C12—H12A	110.8
C4—C3—H3	119.5	C8—C12—H12B	110.8
C2—C3—H3	119.5	C11—C12—H12B	110.8
C3—C4—C5	120.4 (5)	H12A—C12—H12B	108.9
C3—C4—H4	119.8	C10'—C9'—H9'A	111.0
C5—C4—H4	119.8	C10'—C9'—H9'B	111.0
C4—C5—C6	120.9 (4)	H9'A—C9'—H9'B	109.0
C4—C5—N2	120.3 (5)	C9'—C10'—C11'	101.5 (9)
C6—C5—N2	118.8 (5)	C9'—C10'—H10C	111.5
C5—C6—C1	120.0 (5)	C11'—C10'—H10C	111.5
C5—C6—H6	120.0	C9'—C10'—H10D	111.5
C1—C6—H6	120.0	C11'—C10'—H10D	111.5
N1—C7—C1	127.5 (5)	H10C—C10'—H10D	109.3
N1—C7—H7	116.2	C12'—C11'—C10'	107.5 (8)
C1—C7—H7	116.2	C12'—C11'—H11C	110.2
N1—C8—C9	110.3 (8)	C10'—C11'—H11C	110.2
N1—C8—C12	118.6 (11)	C12'—C11'—H11D	110.2
C9—C8—C12	106.7 (8)	C10'—C11'—H11D	110.2
N1—C8—H8A	106.9	H11C—C11'—H11D	108.5
C9—C8—H8A	106.9	C11'—C12'—H12C	110.5
C12—C8—H8A	106.9	C11'—C12'—H12D	110.5
C8—C9—C10	104.9 (8)	H12C—C12'—H12D	108.7

Symmetry codes: (i) −*x*+3/4, −*y*+7/4, *z*.
